# An Innovatory Surgical Technique for Submacular Hemorrhage Displacement by Means of a Bioengineering Perspective

**DOI:** 10.3390/vision5020023

**Published:** 2021-05-18

**Authors:** George Pappas, Nectarios Vidakis, Markos Petousis, Vasiliki Kounali, Apostolos Korlos

**Affiliations:** 1 Ophthalmology Department, General Hospital Venizeleio, 44 Knossou Avenue, 71409 Heraklion, Greece; retinacrete@gmail.com (G.P.); vkounali@gmail.com (V.K.); 2 Mechanical Engineering Department, Hellenic Mediterranean University, Estavromenos, 71004 Heraklion, Greece; vidakis@hmu.gr; 3 Department of Industrial Engineering and Management, International Hellenic University, 14th km Thessaloniki—N. Moudania, Thermi, 57001 Thessaloniki, Greece; apkorlos@ihu.gr

**Keywords:** subretinal hemorrhage (SRH), Age-related Macular Degeneration (AMD), vitrectomy, bubbles coarsening, biphasic absorption, bioengineering

## Abstract

The purpose of this case report is to present a new surgical technique for the treatment of large Subretinal Hemorrhage (SRH) secondary to Age-related Macular Degeneration (AMD). Considering the biomechanics of foam evolution theory, bubble coarsening effect, and gas–liquid biphasic absorption, an SRH due to an AMD case was treated with vitrectomy. The treatment was implemented by subretinal injection of air bubbles combined with rtPA followed by air fluid exchange. The air bubbles helped mess up and remove the blood from the macula area, and no complications occurred. Two weeks postoperatively, there was no sign of hemorrhage and the Central Macular Thickness (CMT) was sharply decreased from 443 μm to 317 μm. At the five-month follow-up, the CMT remained at 267 μm and the patient’s visual acuity improved from light perception to 20/70 according to the Snellen chart. The combination of injecting multiple air bubbles and submacular rtPA, followed by air fluid exchange, was able to displace more than (90%) of the subretinal blood just two weeks postoperatively. Our technique is a promising alternative surgical approach for the displacement of SMH due to AMD, with a clear visual and anatomical benefit seen in the early follow-up period.

## 1. Introduction

Submacular Hemorrhage (SMH) is a vision-threatening condition, especially in older patients. These hemorrhages may result from abnormalities of the retinal or/and choroidal vasculature, such as retina artery macroaneurysm and Choroidal Neovascular Membrane (CNV) secondary to Age-related Macular Degeneration (AMD) [1]. However, it has most often been reported to occur in eyes with Polypoidal Choroidal Vasculopathy (PCV) [2].

When SMH’s are thick and extended, the neurosensory retina can be detached from the supporting Retinal Pigment Epithelial (RPE) layer. This separation in combination with the toxic effect of ferritin, the final metabolite of the dehemoglobinized erythrocytes, can become devastating for the neurosensory retina [3]. The final result is extensive photoreceptor atrophy in the overlying neuroretina and formation of macular scars. The damage is also caused by the accumulation of fibrin as well as clot retraction, which may cause a shearing effect on Photo Receptor (PR) outer segments. SMH may also become a mechanical barrier to metabolic exchange between the RPE and the outer retina [3].

Given the poor natural course of this entity, it is well accepted that visual impairment is time dependent and relates to the underlying disease and previous macular function. The optimal management of patients with SMH is uncertain, and there is no standardized treatment protocol. Several therapeutic approaches have been proposed so far, and the common goal of all is to displace the blood under the macula promptly in order to prevent irreversible photoreceptor damage. These management strategies include Pneumatic Displacement (PD) [4], intravitreal administration of tissue plasminogen activator (tPA) alone or in combination with PD or vitrectomy [5,6], gas displacement alone or combined with subretinal administration of tPA [7,8], and intravitreal injection of anti-VEGF as monotherapy or combined with tPA and PD or vitrectomy [9].

The existing methodologies presented in the literature so far were analyzed, and it was decided that exploiting biomechanics of foam evolution and the bubble coarsening effect could significantly accelerate the therapy process. In the perspective of these theories, a new protocol was developed and evaluated for quicker and more efficient displacement of the hemorrhage blood. After a thorough assessment of the methodology’s risks, the protocol was successfully implemented in a case and presented a lack of complications.

## 2. The Case

**Figure 1:**
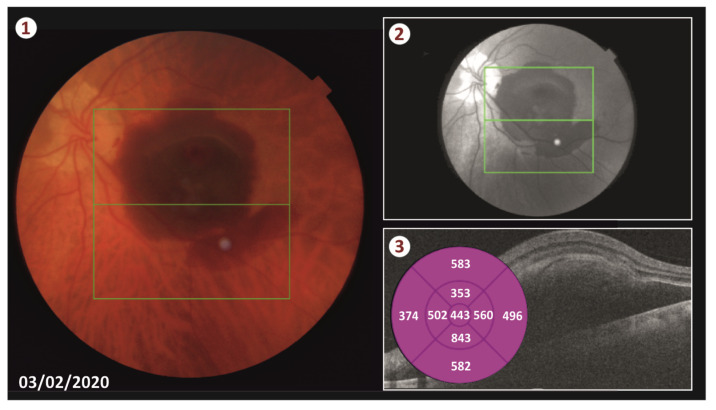
Fundus picture (photos 1 and 2) of the patient’s left eye at first visit. A large submacular hemorrhage due to AMD is shown. The OCT (photo 3) indicates a CMT of 443 μm.

Our patient was a 72-year-old male who, in February 2020, visited the hospital complaining about decreased vision in his left eye for two days. His visual acuity was Light Perception (LP), and his intraocular pressure was within normal limits. He was phakic, and his previews ophthalmic history was unremarkable. The fundoscopy revealed a large submacular hemorrhage due to AMD (Figure 1). We performed an Optical Coherence Tomography (OCT) in order to measure the thickness and the extent of hemorrhage, which was about 3.5 disks in diameter. The patient was treated with vitrectomy, subretinal administration of rtPA, displacement of the blood with gas micro bubbles, and intravitreal anti-VeGF four days after the onset of the symptoms.

## 3. Case Report

An ethics committee approval (number 318032021/28-7) and a patient consent form were obtained for this case. We performed vitrectomy with Alcon CONSTELLATION^®^ Vision system using NGENUITY 3D visualization system (Alcon Laboratories, Geneva, Switzerland). After core vitrectomy, induction of a posterior vitreous detachment was performed. A complete vitrectomy was performed. A 25 g/38 g needle was placed in a 10 mL silicone syringe of the Alcon VFC silicone oil injection system, filled with 0.05 mL of 50 μg rtPA (Actilyse^®^, Boehringer Ingelheim, Ingelheim am Rhein, Germany).

The system was placed in the extract mode, and the foot pedal was activated for a few seconds. This created a minimal airspace in the syringe. Then, the system was placed in the inject mode. The 38 g needle perforated the retina, and the foot pedal was activated. Air bubbles were injected under the retina, and an injection of tPA was performed under the detached neuroretina. The purpose of the bubbles was to create a dense froth within the intensively viscous hematoma, i.e., a biphasic dynamic system, which resolves the blood into a larger volume, absorbs blood microparticles within the bubbles [10], and finally it facilitates the rapid action of rtPA. It is well established by foam dynamics theory that aging of the microbubbles (coarsening effect) forms larger bubbles with gradually lower internal pressure and biphasic thickness, which further progressively collapse and release air molecules within the surrounding retinal tissues [11,12,13].

**Figure 2:**
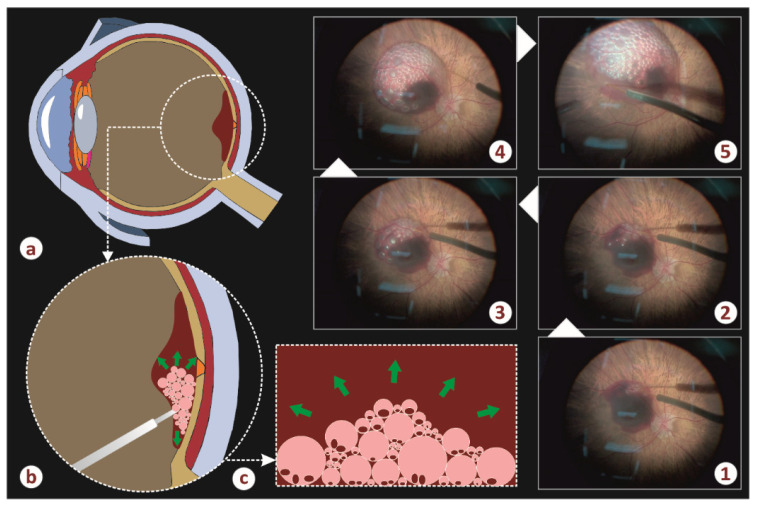
The key steps of the procedure followed. The graphics (**a**–**c**) illustrate a schematic representation of the procedure, while photos 1 to 5 depict the course of the procedure during the surgery, on the phase where air micro-bubbles are injected subretinally.

Figure 2 illustrates the air infusion phase of the implemented operation. More specifically, schemes a–c show the formation and the evolution of the air–blood froth. Five successive steps of the infusion (photos 1–5) are depicted in the same figure. It is evident that the hematoma chamber gradually increases in diameter and height whereas the initial blood spot reduces its dimensions and moves to the vicinity of the chamber.

**Figure 3:**
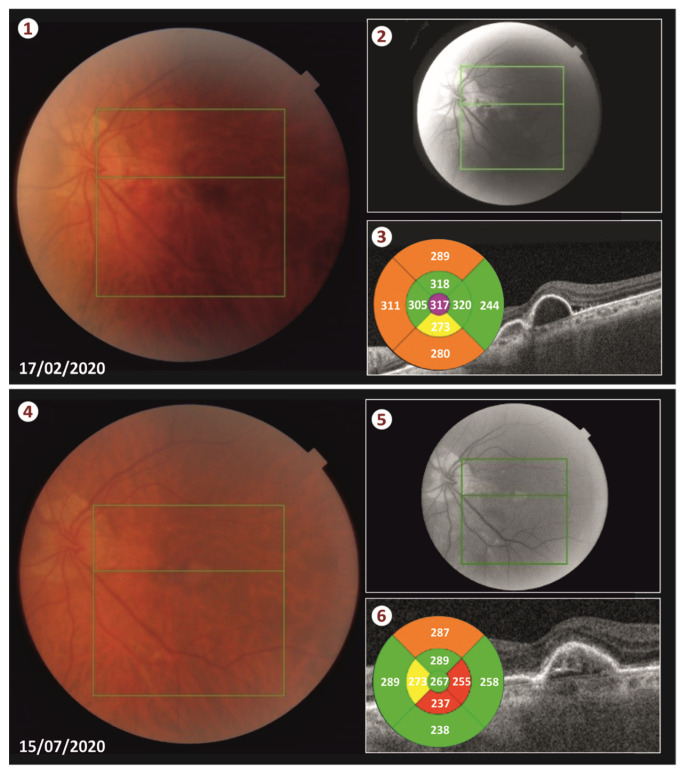
Photos 1 to 3 show the results of the treatment with the proposed procedure twelve days after the surgery, showing an impressive reduction in the CMT, while photos 4 to 6 show the long-term result of the treatment with the proposed procedure about five months after the surgery, indicating a normalization of the patient’s CMT.

The air bubbles helped to remove the blood and created a moving current, which acted constantly under the detached retina and messed up the blood. As a result, we observed blood removal from the submacular area faster. Finally, air/fluid exchange was performed. Intravitreal ranibizumab (Lucentis^®^, Genentech, Inc., South San Francisco, CA, USA) was injected in the eye at the end of the surgery. The duration of the surgery procedure was approximately 25 min. We had the patient lie in a face down position for 48 h. Less than two weeks following the surgery, the blood was totally displaced from the submacular space (see Figure 3 and Video S1).

## 4. Results and Discussion

Subfoveal hemorrhage due to AMD can be a destructive complication, yet there is no large randomized, controlled clinical trial to establish the most effective therapeutic approach. Many different combinations of techniques more or less invasive have been proposed from several authors to treat subfoveal hemorrhages.

This novel technique is, to our knowledge, the first that describes a new combination of maneuvers in order to treat SMH related to AMD. The goal of this approach was to displace the subretinal hemorrhage away from the macula, mechanically, in order to assist the potency of rtPA and to remove the blood safely and faster than existing approaches.

Our patient was successfully treated with this new technique as the blood was totally displaced from the retina and no complications occurred neither during the operation or postoperatively. More specifically, 2 weeks post-surgery, there was no sign of hemorrhage and the patient’s CMT was sharply decreased from 443 μm to 317 μm. We performed three intravitreal injections of ranibizumab (Lucentis) in total. The first one was performed on the day of the surgery, and the following two injections were administrated monthly thereafter. Five months postoperatively, the CMT remains at 267 μm and patient’s visual acuity is 20/70 according to the Snellen chart.

It seems that vitrectomy in combination with subretinal rtPA and air displacement is probably a more effective technique compared to others for removing SMH. Clot liquefaction was enhanced, and less shear stress was applied on the PRs. Additionally, it seems to be a better approach to achieving a faster and more effective displacement of the blood [14].

Subfoveal displacement of the SMH with vitrectomy seems to prevail over the approach with intravitreal gas displacement. Given that, we aimed to maximize the final result by applying the most accepted procedure adapted to a technique with lower surgical morbidity. Specifically, manipulations in the subretinal space may affect the postoperative visual function by inducing photoreceptor and/or RPE trauma. Similarly, the formation of RPE rip after subretinal gas displacement, especially when PEDs are present, has been described by other authors. In our case, there was no surgical injury and no sign of RPE rip detected on the OCT at five-month follow-up with our patient.

During visits, there was a small increase in the height of the PED by about 100 μm, which was overturned after ranibizumab administration, a fact that confirms the beneficial effect of the anti-VeGF injections in the SMH related to AMD. The achievement of our goal is reflected in the anatomical and functional outcome as the patient’s best corrected visual acuity reached (20/70) and the CMT fell to 267 μm according to his last visit (see Figure 3).

The advantages of our technique are that we performed a fast and safe procedure with distinct steps, without any intraoperative complications, achieving a total displacement of the hemorrhage and a satisfactory final outcome.

The limitations are that we present only one case that has been treated with this novel approach, the short-term follow-up, and the fact that the maneuvers of this procedure might need to be performed by experienced vitreoretinal surgeons.

In conclusion, the combination of injecting submacular multiple air bubbles just before submacular rtPA injection followed by air fluid exchange was able to displace more than (90%) of the subretinal blood volume between baseline in just two weeks’ time postoperatively. Our technique seems to be a promising alternative surgical approach for the displacement of SMH due to AMD having a clear visual and anatomical benefit seen in the early follow-up period.

The benefit of using air, compared to a balanced salt solution, subretinally has been addressed in the literature [15]. What we achieved with our technique is the use of air subretinally in a multiple-bubbles fashion. This is a more controlled way of injecting air subretinally. This reduces the major surgical complication described in Sharma et al. [15], which is the creation of a full thickness macular hole and is a potential complication that requires further surgery.

With our technique, which we explained with the laws of physics, we removed the blood from the center of the macula via a smoother method. The bubbles moved the blood in the periphery of the subretinal space created before, and they were keep there when we put the patient in a face down position for a few hours. Even when we put the patient in an upright position, the bubbles did not allow the blood to return to the center. Finally, in our technique, we did not fill the vitreous cavity with SF6 gas, which is absorbed within a six-week period and causes significant handicaps for the patient. We used air, which is absorbed faster, within 2 weeks. In conclusion, our technique is safer and we obtained the final outcome faster and with minimal discomfort for the patient.

## Data Availability

All data generated or analyzed during this study are included in this published article and its Supplementary Materials.

## Supplementary Materials

The following is available online at https://www.mdpi.com/article/10.3390/vision5020023/s1, Video S1: A surgical video presenting the major procedures described in this work: Subretinal Hemorrhage V1.mp4.
